# LKB1/AMPK Pathway and Drug Response in Cancer: A Therapeutic Perspective

**DOI:** 10.1155/2019/8730816

**Published:** 2019-10-31

**Authors:** Francesco Ciccarese, Elisabetta Zulato, Stefano Indraccolo

**Affiliations:** Istituto Oncologico Veneto IOV-IRCCS, Padova, Italy

## Abstract

Inactivating mutations of the tumor suppressor gene Liver Kinase B1 (*LKB1*) are frequently detected in non-small-cell lung cancer (NSCLC) and cervical carcinoma. Moreover, LKB1 expression is epigenetically regulated in several tumor types. LKB1 has an established function in the control of cell metabolism and oxidative stress. Clinical and preclinical studies support a role of LKB1 as a central modifier of cellular response to different stress-inducing drugs, suggesting LKB1 pathway as a highly promising therapeutic target. Loss of LKB1-AMPK signaling confers sensitivity to energy depletion and to redox homeostasis impairment and has been associated with an improved outcome in advanced NSCLC patients treated with chemotherapy. In this review, we provide an overview of the interplay between LKB1 and its downstream targets in cancer and focus on potential therapeutic strategies whose outcome could depend from LKB1.

## 1. Introduction

The Liver Kinase B1 (*LKB1*, also known as *STK11*) is a tumor suppressor gene encoding a ubiquitously expressed and evolutionarily conserved serine threonine kinase, originally associated with the inherited cancer disorder Peutz-Jeghers Syndrome [1, 2]. Inactivating somatic mutations of *LKB1* are frequently reported in non-small-cell lung cancer (NSCLC) [3], malignant melanoma [4], and cervical carcinoma [5]. LKB1 positively regulates the AMP-activated protein kinase (AMPK) [6] and at least 12 additional AMPK-related downstream kinases, involved in the control of cell growth and metabolism and in the regulation of cellular response to energy stress and establishment of cell polarity [7]. Deregulation of LKB1 signaling has been implicated in oncogenesis across many cancer types [8–10], although the energy-sensing function of LKB1-AMPK may also confer a survival advantage under unfavourable conditions [11].

Several preclinical studies identified LKB1 signaling axis as a potential modifier of response of cancer cells to different drugs. Thus, understanding the different mechanisms that account for anti- or prooncogenic effect of LKB1 is essential to identify therapeutic strategies targeting this pathway.

In this review, we address the potential vulnerabilities of LKB1-deficient tumors and focus on recent scientific findings that support a role of this pathway in the modulation of drug response in cancer.

## 2. LKB1 Alterations in Human Cancers

Germline loss of LKB1 kinase activity accounts for the Peutz-Jeghers Syndrome, an autosomal dominant inherited disorder characterized by hamartomatous polyps in the gastrointestinal tract and mucocutaneous pigmentation [2]. Peutz-Jeghers Syndrome is associated with age-related increased risk of cancer development, principally involving the gastrointestinal tract but affecting also the breast, gynecologic tract, lung, and other sites [12], corroborating a *bona fide* tumor suppressor role for LKB1.

**Figure 1:**
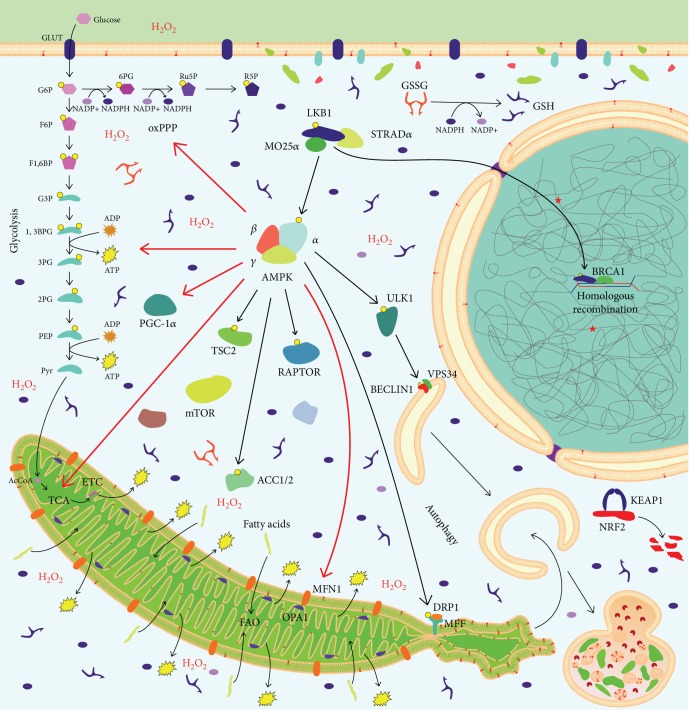
LKB1-proficient tumors display coordinated control of metabolism, DNA repair, and mitochondrial dynamics. LKB1 interacts with the pseudokinase STE20-Related Kinase Adaptor Alpha (STRAD*α*) and with the armadillo-repeat containing protein MO25*α*. Once activated, LKB1 phosphorylates AMPK, which coordinates activation of catabolic processes—such as glycolysis, Krebs cycle, pentose phosphate pathway, fatty acid oxidation, and autophagy—and inhibition of anabolic processes—such as fatty acid synthesis and mTOR pathway. This maximizes ATP production and NADPH regeneration, thus controlling energy and redox homeostasis. Moreover, AMPK promotes mitochondrial fusion and mitophagy of damaged mitochondrial portions. In the nucleus, LKB1 fosters genomic integrity through sustaining homologous recombination. Black arrows from AMPK: direct phosphorylation. Red arrows: activation/upregulation. Yellow circles: phosphate groups. Red phospholipids in membranes: peroxidised phospholipids. Red stars in the nucleus: DNA damage sites. G6P: glucose 6-phosphate; F6P: fructose 6-phosphate; F1,6BP: fructose 1,6-biphosphate; G3P: glyceraldehyde 3-phosphate; 1,3BPG: 1,3-biphosphoglycerate; 3PG: 3-phosphoglycerate; 2PG: 2-phosphoglycerate; PEP: phosphoenolpyruvate; Pyr: pyruvate; AcCoA: acetyl-coA; 6PG: 6-phosphogluconate; Ru5P: ribulose 5-phosphate; R5P: ribose 5-phosphate; GLUT: glucose transporter; GSH: reduced glutathione; GSSG: oxidized glutathione; H_2_O_2_: hydrogen peroxide; oxPPP: oxidative pentose phosphate pathway; TCA: tricarboxylic acid cycle; ETC: electron transport chain; FAO: fatty acid oxidation. The names of proteins deriving from disassembly of mTORC1 and NADPH oxidase complexes are omitted. See the text for details.

In the great majority of human cancers, somatic mutations of the *LKB1* gene are rare. However, *LKB1* is the most frequently mutated gene in cervical carcinoma (20% of cases [5]) and the third most mutated gene in NSCLC (30% of cases in the Caucasian population [13]). Frequent somatic *LKB1* loss in lung adenocarcinoma is puzzling, as lung cancer is uncommon in Peutz-Jeghers patients. In contrast, *LKB1* somatic mutations are rare in colorectal cancer [14], the most frequent neoplasia associated with inherited *LKB1* loss. Several factors could account for these differences. First, *LKB1* loss in NSCLC is frequently homozygous [15], indicating that probably monoallelic LKB1 in Peutz-Jeghers patients is sufficient to limit lung tumorigenesis. Second, *LKB1* mutations coexist with several other genetic alterations in sporadic cancers. *TP53* and *KRAS* are, respectively, the first and the second most mutated genes in lung adenocarcinoma. About 12% of NSCLC cases have *LKB1* and *KRAS* comutations [16]. Moreover, *LKB1* mutations cooccur with gain-of-function *TP53* mutations in 8.2% lung adenocarcinomas [17]. Third, *LKB1* mutations are associated with smoking history of NSCLC patients [18]. Fourth, by interacting with breast cancer susceptibility 1 (BRCA1), LKB1 is involved in the DNA damage response, promoting homologous recombination (Figure 1) and fostering genomic stability [19]. In light of these considerations, *LKB*1 loss could be induced by and, afterwards, facilitate the mutagenic properties of carcinogens contained in tobacco smoke, being selected to promote lung tumorigenesis, while other malignancies—such as colon cancer—have evolved different protumorigenic alterations.

An interesting feature of NSCLC is its intratumor heterogeneity. Remarkably, somatic *LKB1* loss is an intermediate event during lung carcinogenesis, which arises clonally in lung cells with preexisting mutations in initiating drivers, such as *TP53* and *KRAS* [20]. The subclonal nature of *LKB1* highlights how the complexity of cancer genetics might impact on tumor progression and resistance to therapy.

Considering all the genetic and epigenetic events that can affect the *LKB1* gene, the estimated real frequency of *LKB1* alterations in NSCLC is as high as 90% [15], hinting at its fundamental role in lung cancer biology. Moreover, it should be emphasized that the frequency of LKB1 loss in other cancer types could be underestimated, due to rarely investigated epigenetic alterations. A paradigmatic example is breast cancer, whose aggressiveness and metastasis are promoted by LKB1 loss [9], even if *LKB1* mutations are detected with low frequency. The combination of sequencing and analysis of protein expression might overcome intrinsic limitations of sequencing and provide a comprehensive evaluation of LKB1 status in tumors.

## 3. Role of LKB1-AMPK Pathway in Cell Metabolism

LKB1 was identified as the critical upstream kinase required for AMPK activation [6, 21, 22] (Figure 1), thus providing a direct link between a known tumor suppressor and regulation of metabolism [23]. AMPK has a central role in the regulation of energy metabolism in eukaryotes and coordinates glucose and lipid metabolism in response to alterations in nutrients and intracellular energy levels, contributing to maintain steady-state levels of intracellular ATP [24].

Upon changes in energy availability, causing perturbations in the ATP-to-ADP or ATP-to-AMP ratio, AMPK is activated by an allosteric mechanism and by LKB1 via phosphorylation [7]. AMPK is also activated by increases in intracellular Ca^2+^ [25–27] and by DNA damage [28–30]. Moreover, a novel AMP-independent mechanism of AMPK activation under glucose starvation has recently been described by Zhang and colleagues who observed that, upon glucose starvation and the consequent decrease of fructose-1,6-bisphosphate (FBP) levels, aldolases promote the formation of a lysosomal complex containing v-ATPase, Ragulator, AXIN/LKB1, and AMPK [31], leading to LKB1-mediated AMPK activation before energy levels fall. This aldolase-dependent mechanism of AMPK activation could be at play under conditions where low glucose does not cause an increase of intracellular AMP-to-ATP or ADP-to-ATP ratios.

Once activated, AMPK redirects metabolism towards decreased anabolism and increased catabolism by phosphorylation of key proteins involved in several metabolic pathways [24], including lipid homeostasis, glycolysis, protein synthesis, and mitochondrial homeostasis.

AMPK was originally defined as the critical inhibitory upstream kinase for the metabolic enzymes acetyl-CoA carboxylase (ACC1 and ACC2) [32] (Figure 1) and HMG-CoA reductase [33], which serve as rate-limiting steps for fatty acid and sterol synthesis, respectively, in a wide variety of eukaryotes. Moreover, inactivation of ACC2 switches on fatty acid (FA) *β*-oxidation in mitochondria [34]. Through activation of FA oxidation and inhibition of FA synthesis, LKB1-AMPK pathway plays a pivotal role in the maintenance of intracellular NADPH levels, which is required to prevent oxidative stress and to promote cancer cell survival under energy stress conditions [35].

Moreover, when nutrient levels are low, AMPK acts as a metabolic checkpoint inhibitor of cell growth, by modulation of the master regulator of growth, the mammalian target of rapamycin (mTOR) pathway [36] (Figure 1). AMPK activation leads to inhibition of mTOR complex 1 (mTORC1), by activation of the negative mTORC1 regulator TSC2 and by inhibition of the mTORC1 subunit RAPTOR [36]. Importantly, activated mTORC1 is localized on the surface of lysosomes, where it is negatively regulated by AXIN through inhibition of the GEF (guanine nucleotide exchange factor) activity of Ragulator. Thus, AXIN/LKB1 complex inhibits mTORC1 through the glucose-sensing mechanism involving aldolase and FBP [31]. Moreover, AMPK activation caused G1 cell cycle arrest associated with activation of p53, followed by induction of the cell cycle inhibition protein p21 and by stabilization via phosphorylation of the cyclin-dependent kinase inhibitor p27^kip1^ [37, 38]. Through mTOR inhibition, AMPK downregulates hypoxia-inducible factor 1*α* (HIF-1*α*), thus counteracting the Warburg effect [39].

In addition to its central role in the regulation of cell growth, mTORC1 controls autophagy, a lysosome-dependent catabolic program that maintains cellular homeostasis. Upon nutrient starvation, mTORC1 is inactivated through the energy-sensing mechanism of AMPK activation. Moreover, mTORC1 is also inhibited by direct dissociation from lysosomes through the glucose-sensing mechanism [31]. This mTORC1 suppression relieves the inhibitory phosphorylation on Unc-51-Like Autophagy Activating Kinase 1 (ULK1), a kinase essential for autophagy induction [40, 41]. AMPK has also an important role in the regulation of autophagy through direct phosphorylation of ULK1 and of a second autophagy-initiating regulator, the lipid kinase complex PI32KC3/VPS34 [42]. Interestingly, AMPK triggers acute destruction of dysfunctional mitochondria through ULK1-dependent stimulation of mitophagy (Figure 1), and it stimulates *de novo* mitochondrial biogenesis through peroxisome proliferator-activated receptor gamma coactivator 1 *α*- (PGC-1*α*-) dependent transcription [38]. Interestingly, genetic deletion of *Lkb1* in the haematopoietic stem cell resulted in mitochondrial dysfunction and deregulation of bioenergetic processes through AMPK-dependent and independent mechanisms [43–45]. The interplay between AMPK and mitochondria is further discussed in a distinct section.

Besides AMPK, other 12 kinases, collectively termed AMPK-related kinases, are LKB1 substrates. However, little is known about what stimuli direct LKB1 towards any of these AMPK-related kinases. These enzymes include two family members, SNARK/Nuak2 and SIK2, both activated under low energy conditions, although only AMPK is activated under low ATP levels [36]. Moreover, other members, such as isoforms of PAR1/MARK, as well as SAD/BRSK, unlike AMPK, are not activated by energy stress but have been implicated in controlling cell polarity [46].

### 3.1. LKB1: An Unexpected Oncogenic Role for a Tumor Suppressor

Recently, the role of LKB1-AMPK to sense different types of stress has pointed at a conditional oncogenic role of this pathway. In fact, its ability to modulate cell metabolism in order to restore homeostasis may confer a survival advantage under selective pressure, by favoring adaptation to hostile conditions [47]. In this context, Lee and colleagues demonstrated that polyubiquitination of LKB1 by S-Phase Kinase-Associated Protein 2 (Skp2) ubiquitin ligase promotes its persistent activation, leading to cell survival and poor outcome in hepatocellular carcinoma patients [48]. A recent study showed that, although it negatively regulates the epithelial-to-mesenchymal transition (EMT)-inducing gene *ZEB1*, LKB1 expression is increased in spheroids obtained from breast cancer cell lines and its ablation induces anoikis, suggesting that LKB1 promotes survival of circulating tumor cells [49]. LKB1 activation can result in an oncogenic program based on the contextual oncogenic role of its targets. For instance, LKB1 upregulates the expression of miR-34a [50], which was found to promote survival in the context of adult T-cell leukemia/lymphoma (ATLL) [51].

Downstream of LKB1, also AMPK has been indicated as a contextual oncogene. In fact, AMPK activation promotes glioblastoma growth by inducing lipid internalization [52] and sustains bioenergetics of glioblastoma through HIF-1*α* signaling [53]. Moreover, AMPK activation results in increased AKT oncogenic signaling through Skp2 phosphorylation under stress [54] and promotes aberrant expression of PGC-1*β* and estrogen-related receptor *α* (ERR*α*) in colon cancer, supporting its survival [55]. Finally, AMPK activation promotes resistance of cancer cells to chemotherapy by induction of autophagy [56–59].

How can the contrasting role of LKB1 as a tumor suppressor or promoter of cancer survival be reconciled? It must be considered that this pathway has evolved to allow cell survival under energy stress. During the initial phases of tumorigenesis, stress is a critical event that alters cell physiology and induces genetic aberrations, genomic instability, and transformation. In this context, LKB1 and AMPK play a tumor suppressor role by dealing with metabolic stress. The maintenance of genomic integrity, activation of autophagy, which scavenges damaged organelles and proteins, and activation of TP53 [14] to eliminate aberrant cells blunt cancer initiation. However, stress is a double-edged sword in cancer, and if not solved, it would lead to tumor eradication. In this scenario, a functional LKB1-AMPK pathway is advantageous for growing cancer cells, as it promotes adaption to a hostile microenvironment and cell survival. The activation of catabolic pathways and increased recycling of cellular components through autophagy ensure maintenance of energy homeostasis [60]. Autophagy, which has both prosurvival and prodeath effects, is probably the main responsible for contextual tumor suppressor and oncogenic activities of LKB1-AMPK. It should be pointed out, however, that autophagic cell death is a concept that should be cautiously evaluated. Cell death occurs, likely, despite autophagy, rather than because of autophagy [61]. In fact, increased autophagy in dying cells could be a rescue mechanism that failed or a mechanism sustaining apoptosis through ATP production. Physiologic “tumor suppressor” autophagy, which degrades damaged organelles and suppresses tumor initiation, should be distinguished by aberrant “prosurvival” autophagy, which is coopted by cancer to sustain its growth. As degradation of cellular components that have been damaged by anticancer therapies is a widely adopted mechanism of resistance, activation of autophagy by LKB1-AMPK in advanced stage cancers could represent a rescue mechanism.

## 4. Mitochondrial Dynamics Is Affected by LKB1-AMPK Pathway

**Figure 2:**
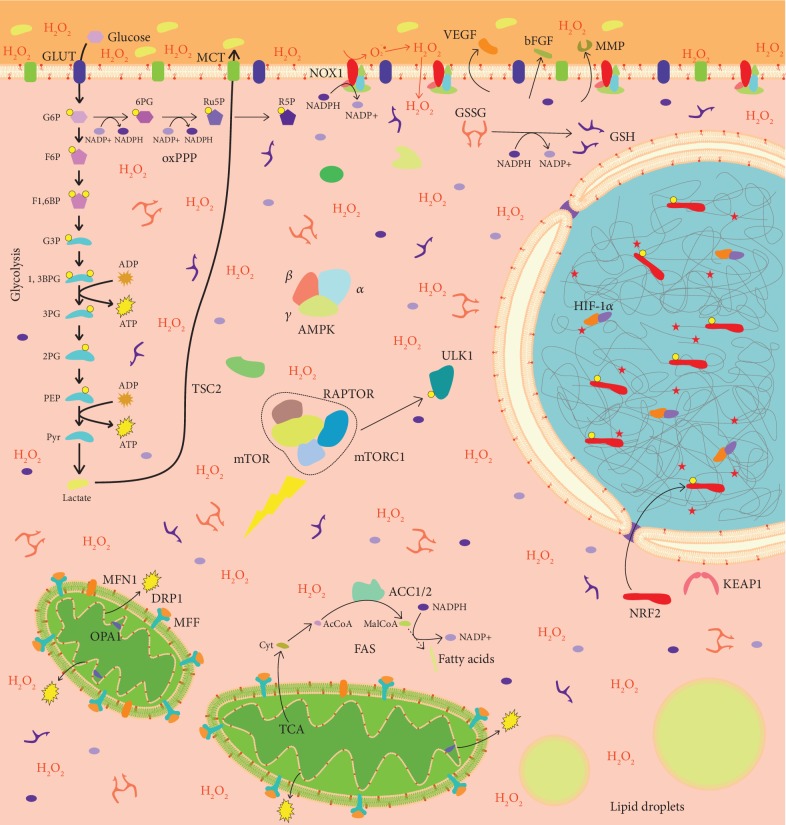
LKB1 loss alters cancer cell biology. LBK1 loss and consequent lack of AMPK activation lead to mTORC1 assembly, resulting in autophagy inhibition. High metabolic requirements imposed by sustained proliferation are met through aerobic glycolysis (i.e., Warburg effect), driven by HIF-1*α* stabilization, which provides cancer cells with ATP and intermediates for anabolic reactions (not shown). Pyruvate is preferentially converted to lactate, which is excreted in the tumor microenvironment. Activation of ACC1 and ACC2 promotes fatty acid synthesis in the cytosol, by using citrate coming from mitochondria. NOX1 expression drives the assembly of NADPH oxidase complex, which produces ROS in the microenvironment. NOX-produced ROS enter the cell, thus inducing oxidative stress and activating NRF2 through the oxidation of KEAP1. Reduced expression of MFN1 and OPA1 and increased activity of DRP1, induced by HIF-1*α* activation, lead to mitochondrial fragmentation. Increased ROS levels and mitochondrial fission promote the secretion of proangiogenic factors in the microenvironment. Yellow circles: phosphate groups. Red phospholipids in membranes: peroxidised phospholipids. Red stars in the nucleus: DNA damage sites. G6P: glucose 6-phosphate; F6P: fructose 6-phosphate; F1,6BP: fructose 1,6-biphosphate; G3P: glyceraldehyde 3-phosphate; 1,3BPG: 1,3-biphosphoglycerate; 3PG: 3-phosphoglycerate; 2PG: 2-phosphoglycerate; PEP: phosphoenolpyruvate; Pyr: pyruvate; 6PG: 6-phosphogluconate; Ru5P: ribulose 5-phosphate; R5P: ribose 5-phosphate; AcCoA: acetyl-coA; MalCoA: malonyl-coA; Cit: citrate; GLUT: glucose transporter; MCT: monocarboxylate transporter; GSH: reduced glutathione; GSSG: oxidized glutathione; H_2_O_2_: hydrogen peroxide; oxPPP: oxidative pentose phosphate pathway; TCA: tricarboxylic acid cycle; FAS: fatty acid synthesis. mTORC1 targets are omitted. See the text for details.

As master regulators of metabolism, LKB1 and AMPK are tightly intertwined with mitochondrial function and dynamics (Figure 1). Mitochondria are essential dynamic organelles that continuously shift from fusion to fission and vice versa. Mitochondrial dynamics is in part regulated by the LKB1-AMPK pathway (Table 1). Following stress, AMPK activates mitochondrial fusion to restore the function of damaged mitochondria. If the damage is too extensive, AMPK activates mitochondrial fission and mitophagy to separate and degrade damaged mitochondrial portions and promotes synthesis of new mitochondria, in order to preserve mitochondrial network function and maximize ATP production (Table 1). In contrast, in LKB1 defective tumors, hypoxic stress elicits activation of HIF-1*α* [62], which reduces the expression of Mitofusin-1 (MFN1) and Optic Atrophy 1 (OPA1) and increases activity of Dynamin-Related Protein 1 (DRP1), thus unbalancing mitochondrial dynamics towards fission (Figure 2). In endothelial cells, this promotes migration, invasion, and tube formation, implying that hypoxia-induced mitochondrial fission activates angiogenesis [63].

Mitochondria fusion and fission are both involved in the response of cancer cells to therapies. Several studies observed that mitochondrial fission sensitizes cancer cells to chemotherapy. Inhibition of autophagy has been shown to enhance doxorubicin cytotoxicity in breast cancer cells through mitochondrial translocation of DRP1 and consequent mitochondrial fission [64]. Similarly, LKB1-deficient NSCLC cell line A549 resulted resistant to doxorubicin-induced apoptotic cell death due to dysfunctional DRP1 that impedes mitochondrial fission [65]. Notably, AMPK promotes the maintenance of mitochondrial membrane potential following stress [66], thus preventing the proteolytic cleavage of OPA1, which is involved in cell death induction [67].

In cancer cells, mitochondrial fission has also been described to trigger cell migration, leading to cell escape from stressful conditions, such as chemotherapy, metastasis, and chemoresistance. By decreasing reactive oxygen species (ROS) levels—as described later—AMPK inhibits the release of high mobility group box 1 (HMGB1), which is involved in mitochondrial fission [68], thus blunting these escape mechanisms.

## 5. Targeting the LKB1-AMPK Pathway

### 5.1. Activation of LKB1-AMPK Pathway by Biguanides

The biguanide metformin attracted considerable attention as a potential anticancer drug once the connection between LKB1 and AMPK was discovered [42]. Metformin is one of the most widely used type 2 diabetes drug worldwide, and epidemiological studies revealed that diabetic patients taking metformin show a statistically significant reduced tumor incidence [69].

Metformin and the related drug phenformin have been shown to inhibit complex I of the mitochondria [70], resulting in increased intracellular AMP and ADP levels, which trigger LKB1-dependent phosphorylation of AMPK [42]. Diabetic patients taking biguanides might have a lower incidence of cancer because of the role of the LKB1-AMPK pathway as a checkpoint inhibitor of cell growth and suppression of mTORC1 and other growth pathways. In addition, antitumor effects of metformin might be linked to its ability to lower circulating blood glucose and insulin levels, which also contribute to cancer risk and incidence in some contexts [69].

Tumor cells lacking functional LKB1 are acutely sensitive to metabolic stress, resulting in rapid apoptosis, likely a consequence of their inability to sense energy stress and activate mechanisms to restore energy homeostasis [6]. Taking advantage of these observations, Shackelford and colleagues tested the therapeutic potential of phenformin in *LKB1*-deficient NSCLC experimental tumors. Phenformin as a single agent reduced tumor burden in *KRAS*/*LKB1* comutated murine NSCLC. In particular, LKB1 inactivation renders NSCLC cells unable to modulate anabolic processes in conditions of metabolic stress caused by phenformin. The constitutive activation of KRAS pathway forced cells to duplicate their DNA and other intracellular structures, thus accelerating energy depletion and damage to intracellular components and triggering apoptosis [71].

In a recent study, it has been speculated that the metabolic frailty of *KRAS*/*LKB1* comutated NSCLC cells could be exploited pharmacologically by the combination of metformin with compounds that increase intracellular stress by interfering with DNA replication and repair, such as platinum compounds [72]. Metformin has been demonstrated to induce apoptosis in *KRAS*/*LKB1* comutated experimental tumors. On the contrary, in *KRAS*^wt^/*LKB1*^wt^ cells or in the *KRAS^mut^*/*LKB1*^wt^ experimental tumors, metformin determined activation of the LKB1/AMPK signaling pathway, thus reducing cell proliferation and metabolic requirements and preventing metabolic crisis in cancer cells. Treatment with metformin was also associated with enhanced cisplatin-induced *in vitro* proapoptotic and *in vivo* antitumor effects specifically in *KRAS*/*LKB1* comutated tumors [72].

The opportunity to target dysregulated metabolic features in *LKB1* mutated tumors could represent a strategy to improve therapeutic efficacy of other compounds affecting cell metabolism. In this regard, stable upregulation of glycolysis in tumor cells has been observed following antiangiogenic treatment [73], and as a master regulator of tumor cell metabolism and tumor microenvironment, LKB1/AMPK has a role in tumor response to VEGF neutralization [74]. Thus, sequential or simultaneous combination of antiangiogenic drugs and metformin might represent a new treatment opportunity for LKB1-deficient tumors. Although clinical and preclinical data are fragmentary, a case of a terminally ill patient with advanced endometrial cancer, showing radiological response to simultaneous administration of metformin and bevacizumab, was described by our group [75]. Interestingly, the high expression of MCT4—a marker of enhanced glycolysis—and loss of LKB1 expression were detected in the patient's liver metastasis sample. These findings suggest that metformin could modulate bevacizumab activity in tumors lacking LKB1 expression and deserves further validation in preclinical studies and clinical trials.

As previously described, autophagy represents a cellular process directed to preserve cellular homeostasis. Complementary with aforementioned findings, the ability to sense and counteract different types of stresses of LKB1 proficient tumor cells might be targeted by the combination of AMPK activators, such as metformin, and autophagy inhibitors, such as chloroquine, which has been recently repurposed as an anticancer agent [76]. Speculatively, this combination, currently evaluated in clinical trials [77], should potentiate the tumor suppressor activity of LKB1-AMPK by inhibiting its oncogenic prosurvival activity.

### 5.2. Targeting the Downstream Effectors of LKB1 Pathway

#### 5.2.1. Inhibition of mTOR

Since LKB1 inactivation promotes mTORC1 signaling [46, 78] (Figure 2), mTOR inhibitors have been extensively tested as a therapeutic approach to target *LKB1* mutated tumors. However, preclinical studies produced controversial results. LKB1 inactivation in endometrial cancers resulted in high responsiveness to mTOR inhibitors [79], and rapamycin monotherapy (mTORC1 inhibitor) decreased polyp burden and size in LKB1^+/−^ mice with polyposis [62]. In contrast, *LKB1* gene inactivation in NSCLC cells did not increase sensitivity to mTORC1 inhibitors, through negative feedback activation of AKT [80]. The same mechanism of escape to rapamycin could be at play in *Lkb1*-inactivated lung adenocarcinoma mouse model [81]. On the other hand, simultaneous inhibition of mTOR and glycolysis was significantly effective at reducing tumor volume and burden in a mouse model of spontaneous breast cancer promoted by loss of LKB1 in an ErbB2 activated model [82]. Given the master regulatory role of mTOR signaling in cell growth, additional preclinical and clinical studies are required in order to establish the appropriate genetic and molecular setting that could influence response to inhibition of mTOR pathway in the context of LKB1 status.

#### 5.2.2. Inhibition of ACC Activity

*De novo* FA synthesis is essential to sustain rapid tumor growth, and reprogramming of lipid metabolism is a newly recognized hallmark of malignancy. Targeting altered lipid metabolic pathways has become a promising anticancer strategy [83]. Lipid-lowering drugs are being considered for clinical trials, showing their advantages in comparison with other anticancer drugs with high toxicity [83]. Since AMPK inhibits activity of ACC [32], the rate-limiting enzyme required for *de novo* FA synthesis, the latter might represent a potential metabolic target in tumors lacking LKB1. Inactivation of LKB1 in the adenocarcinoma mouse model determined accumulation of lipids and low levels of FA oxidation signature genes [81]. In preclinical models, ACC was required to maintain *de novo* FA synthesis needed for growth and viability of NSCLC cells, and its pharmacological inhibition results in robust inhibition of tumor growth [84]. Administration of ND-646—an allosteric inhibitor of the ACC enzymes ACC1 and ACC2 that prevents ACC subunit dimerization—as a single agent or in combination with the standard-of-care drug carboplatin markedly suppressed lung tumor growth in NSCLC xenograft from LKB1-deficient cells [84]. Effects of ACC inhibition on tumor growth fit its critical role in maintaining *de novo* FA synthesis and prompt further investigation to define new strategies to target LKB1-defective tumors.

### 5.3. Role of LKB1 in response to Therapy-Induced Oxidative Stress

ROS are signaling molecules that regulate several biological processes—such as autophagy, immunity, and differentiation—through reversible thiol oxidation [85]. On the other hand, excessive ROS levels induce irreversible modification of proteins, alongside with oxidation of lipids and nucleic acids, thus leading to oxidative stress and cell death [86]. Cell fate (i.e., growth arrest, proliferation, or death) is hypothetically decided by a ROS rheostat [87], which, in cancer cells, is set to intermediate levels to sustain tumor growth. A further increase in ROS levels induces extensive damage to cell structures and selective elimination of cancer cells, implying modulation of redox homeostasis as a promising anticancer strategy [88]. Several chemotherapeutic agents and radiotherapy, indeed, kill cancer cells by increasing ROS levels beyond the toxic threshold. Cisplatin [89], paclitaxel and other taxanes [90], doxorubicin [91], cytarabine [92], and arsenic trioxide [93] are some examples of traditional drugs that induce lethal oxidative stress in cancer cells. Moreover, several mitochondria-targeting compounds, such as capsaicin [94], betulinic acid [95], and curcumin [96], induce cancer cell death by increasing ROS levels.

Several studies reported that LKB1-AMPK pathway is involved in the maintenance of redox homeostasis by contrasting ROS production and promoting ROS scavenging (Figure 1). Following metabolic stress, AMPK inhibits NADPH-consuming FA synthesis and increases NADPH-producing FA oxidation, thus maintaining elevated levels of NADPH, the universal electron donor used to regenerate ROS scavenging systems, leading to cancer cell survival [35]. ROS are able to activate AMPK, which, in turn, lowers ROS levels by inducing PGC-1*α*-mediated antioxidant response [97]. In response to ROS, AMPK activation also promotes glycolysis and pentose phosphate pathway (PPP), thus increasing NADPH levels [98]. Recently, it has been found that the mitochondrial NADPH pool is maintained by pathways other than the PPP [99]. AMPK activates Sirtuin-3 (SIRT3), which deacetylases isocitrate dehydrogenase 2 (IDH2), one of the principal contributors to NADPH production in mitochondria, thus increasing its activity [100]. Moreover, by increasing the activity of the tricarboxylic acid cycle and FA oxidation [7], AMPK could contribute to NADPH production in mitochondria through IDH2 and malic enzymes (ME) 2 and 3. LKB1 regulates oxidative stress response through p38-mediated upregulation of mitochondrial superoxide dismutase 2 (SOD2) and catalase, which scavenge ROS [101].

Given the established role of LKB1 and AMPK in maintaining redox homeostasis and the ability of ROS to kill cancer cells, one can speculate that functional LKB1-AMPK pathway could be a negative predictor of response to ROS-inducing therapies. Several evidences suggest that this is, in fact, the case.

**Figure 3:**
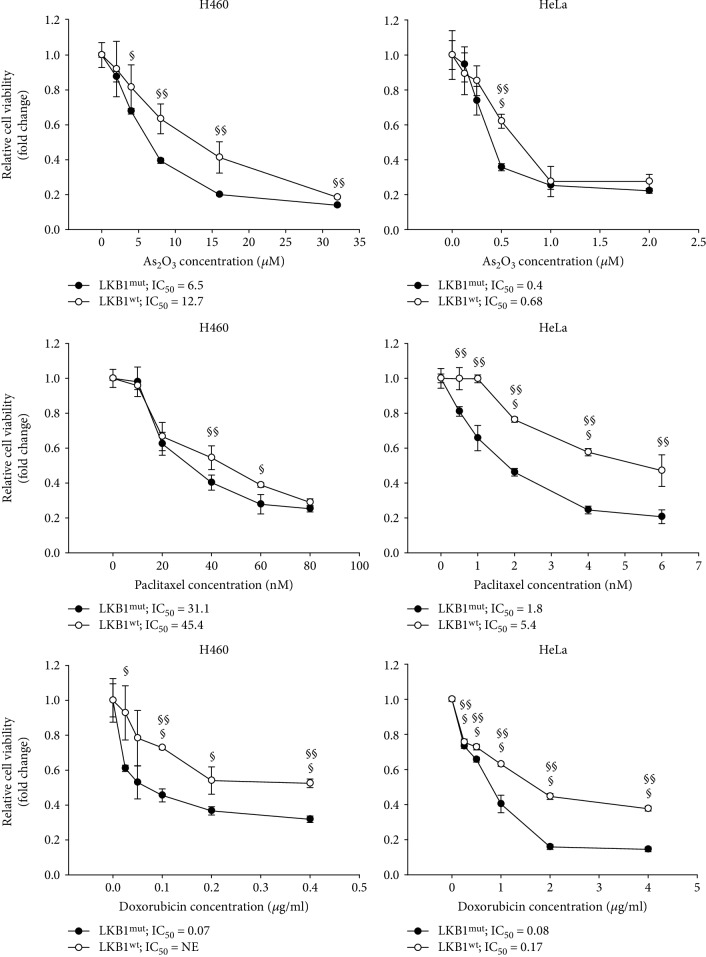
LKB1 expression regulates response to oxidative stress induced by prooxidant cytotoxic drugs. Isogenic pairs of H460 and HeLa cells (derived from NSCLC and cervical carcinoma, respectively) differing in LKB1 status and generated as described by Zulato et al. [103] were treated with arsenic trioxide, paclitaxel, or doxorubicin for 48 h. Viability was evaluated by the Sulphorhodamine B assay (for materials and methods, refer to [103]) in cells exposed to increasing concentrations of drugs. For each cell line tested, the IC_50_ values relative to LKB1^mut^ and LKB1^wt^ cells are reported. Results are representative of three independent experiments performed in triplicate (^§^*P* < 0.05, ^§§^*P* < 0.01, and ^§§§^*P* < 0.001 LKB1^wt^ versus LKB1^mut^ cells). Results of SRB assay revealed that H460 and HeLa LKB1^wt^ variants were more resistant than their LKB1^mut^ counterparts to the drugs tested. NE: not evaluable.

In our recent work, we observed that LKB1 loss in NSCLC cells is associated with the increased expression of NADPH oxidase 1 (NOX1), leading to elevation of ROS levels (Figure 2) and exacerbated sensitivity to exogenous oxidative stress [102]. Preliminary results by our group indicate that LKB1 deficiency is associated with increased response to several ROS-inducing drugs commonly used in the clinic, such as arsenic trioxide, paclitaxel, and doxorubicin (Figure 3), thus suggesting that LKB1 status could predict tumor response to several chemotherapeutic regimens. Moreover, we found that LKB1-defective cancer cells undergo a decrease in reduced glutathione levels following exogenous oxidative stress and are more sensitive to cisplatin and *γ*-irradiation, compared with LKB1-proficient cancer cells. LKB1-defective NSCLC cells exposed to exogenous oxidative stress lose their mitochondrial membrane potential and undergo mitochondrial fragmentation, while LKB1-proficient cancer cells maintained polarized and fused mitochondria [103]. These results imply that LKB1-AMPK pathway exerts a protective effect towards oxidative stress, blunting the efficacy of ROS-inducing therapies. Remarkably, low-null LKB1 expression by IHC was retrospectively associated with the improved outcome in advanced NSCLC patients treated with first-line platinum-based chemotherapy [104]. This finding may be explained by considering the well-established role of LKB1 as a genomic sensor participating in the DNA damage response triggered by oxygen radicals. Consistently, LKB1-defective cells exposed to exogenous oxidative stress showed extensive macromolecular damage, measured as membrane lipid peroxidation, accumulation of nucleic acid oxidation marker 8-oxoguanine in mitochondrial DNA, and accumulation of DNA damage marker phosphorylated histone 2AX (*γ*H2AX). Strikingly, LKB1-defective cells demonstrated oxidation of mitochondrial DNA even under basal culture conditions, alongside with more fragmented mitochondria compared to LKB1-proficient cells. These findings support that LKB1 and AMPK protect cells from excessive oxidation of lipids and nucleic acids both by decreasing NOX-mediated ROS production and by increasing ROS scavenging, thus blunting the efficacy of anticancer therapies aimed at impairing redox homeostasis. In line with our findings, Li and colleagues observed that LKB1 loss in lung adenocarcinoma is associated with increased ROS levels, which drive cancer plasticity and drug resistance through transdifferentiation to squamous cell carcinoma in the *KRAS*-*LKB1*- (KL-) mutant lung cancer mouse model [81]. Squamous cell carcinoma, compared to adenocarcinoma, upregulated the expression of genes involved in the metabolism of glutathione and of NRF2 target genes, thus reducing DNA oxidation. Interestingly, Li and colleagues observed an inverse correlation between LKB1 expression and 8-oxoguanine levels in human NSCLC, where a proportion of cells with LKB1 loss and high 8-oxoguanine staining expressed squamous cell carcinoma markers. Reexpression of AMPK in the KL adenocarcinoma model decreased ROS levels and DNA oxidation by increasing FA oxidation-derived NADPH production, indicating the involvement of AMPK in LKB1-mediated ROS decrease, according to our findings [103]. Interestingly, Li and colleagues observed that treatment with phenformin in KL model resulted in the selective survival of squamous cell carcinoma clones and in transdifferentiation of adenocarcinoma to squamous cell carcinoma. Findings from Li and colleagues imply that LKB1 loss in adenocarcinoma could select for clones resistant to oxidative stress through increased activity of the transcription factor NRF2. Interestingly, KEAP1 is frequently inactivated in NSCLC (about 20% of cases [105]), and LKB1-defective tumors have more than sixfold increased odds of bearing KEAP1 loss compared to LKB1-proficient cancers [106]. Consequently, LKB1 loss is frequently associated with aberrant activation of NRF2 pathway, which drives aggressiveness and resistance to therapy. Constitutive NRF2 activation in cancer is connected with transcriptional programs aimed at increasing NADPH and glutathione levels, such as the serine synthesis pathway [107], which fuels mitochondrial folate cycle, the principal contributor to NADPH production in cells [99]. Thus, constitutive NRF2 activation is frequently coselected with LKB1 loss in human cancers to compensate for increased oxidative stress induced by lack of AMPK activation.

### 5.4. Role of LKB1-AMPK in Therapy-Induced Senescence

Different types of stress, such as oxidative or oncogenic stresses, can induce an irreversible cell cycle arrest. Permanent blockade of cell proliferation, known as senescence, is a valuable anticancer strategy that could be achieved through sublethal chemotherapy and irradiation. High doses of chemotherapeutics or radiation cause massive damage to cell structures, leading to cell death not only in cancer cells but also in highly proliferating normal cells. On the contrary, low doses of anticancer drugs or radiation lead to therapy-induced senescence (TIS) only in cancer cells, thus decreasing side effects [108]. Noteworthily, several chemotherapeutics, including cisplatin, doxorubicin, etoposide, and resveratrol, induce senescence in cancer cells [109].

Contrasting data regarding the role of AMPK on senescence induction are reported in the literature. As oxidative stress is a senescence inducer and AMPK is involved in the maintenance of redox homeostasis, it is not surprising that LKB1-AMPK pathway could prevent senescence in cancer cells [110]. Han and colleagues observed that hydrogen peroxide-induced senescence is associated with inhibition of AMPK. Furthermore, pharmacological activation of AMPK prevented the induction of senescence by oxidative stress, through restoration of autophagy. Interestingly, the authors observed that inhibition of autophagy through chloroquine aggravated senescence induced by hydrogen peroxide and blunted the protective role of AMPK activation. Moreover, NAD^+^ levels are decreased in senescent cells as a consequence of NAD^+^ salvage pathway reduction and increased NAD^+^ consumption by PARP-1. Pharmacological activation of AMPK promoted synthesis of NAD^+^ through salvage pathway, thus increasing the activity of NAD^+^-consumer SIRT1, which positively regulates autophagy. The results from Han and colleagues have important implications for cancer therapy. First, AMPK could have a protective role against TIS when the latter arises from a chemotherapeutic regimen that triggers oxidative stress. In this regard, metformin could increase the efficacy of chemotherapy, as described above, but could impair TIS, thus favouring the burden of surviving cells and tumor relapse. Second, autophagy emerges as an important escape mechanism from TIS, confirming its central role in the oncogenic properties of LKB1-AMPK pathway. The use of chloroquine or other inhibitors of lysosomal acidification in the clinic should enhance TIS, thus achieving remarkable anticancer activity.

On the other hand, the activation of SIRT1 and AMPK has been associated with the induction of senescence in colorectal carcinoma cells [111]. Jung and colleagues observed that aspirin induced senescence in two colorectal carcinoma cell lines, but not in normal colonic cells, through the increased expression and deacetylase activity of SIRT1 and the increased activation of AMPK. The enhanced activity of SIRT1 and AMPK was induced by a decrease of ATP levels in aspirin-treated cancer cells, as observed with irradiation. Interestingly, the authors demonstrated that knockdown of SIRT1 or inhibition of its deacetylase activity decreased aspirin-induced and irradiation-induced senescence. The same results were obtained through knockdown or inhibition of AMPK. On the contrary, activation of SIRT1 through resveratrol or of AMPK through AICAR promoted the induction of senescence. The data from Jung and colleagues are consistent with the known senescence-inducing activity of resveratrol. Thus, it is reasonable that in certain cellular contexts SIRT1 and AMPK induce senescence rather than inhibit it, as observed by Han and colleagues. The decreased levels of ATP observed in aspirin-treated cells, however, suggest that in this context autophagy could not play a central role. Although aspirin induces autophagy [112], it is possible that the latter was a rescue mechanism only in the context described by Han et al., thus profoundly altering the outcome of AMPK activation. The positive role of LKB1-AMPK pathway on senescence is supported by different studies. Yi and colleagues observed that low doses of metformin induced senescence of hepatoma cells through activation of AMPK [113]. Metformin also induced the acetylation of p53 as a consequence of AMPK-mediated inhibition of SIRT1 deacetylase activity on p53. Similarly, Liao and colleagues demonstrated that AMPK activation is involved in the metabolic alterations associated with radiation-induced senescence [114].

In conclusion, AMPK positively regulates TIS, implying that LKB1-proficient tumors could be more susceptible to a radiochemotherapeutic regimen that induces senescence. It should be considered, however, that AMPK-induced autophagy could be an escape mechanism that impairs TIS, thus curbing the efficacy of anticancer treatments. In this regard, a recent study provides evidence for a role of AMPK as a predictive factor of response to senescence-inducing therapies. In fact, Wang and colleagues observed that trametinib radiosensitized LKB1-defective NSCLC cells, while LKB1-proficient cells were protected by senescence through AMPK-mediated autophagy [115]. The central role of autophagy as a rescue mechanism—as recently confirmed by the observation of autophagy-mediated protumorigenic effects in the context of mitotic slippage-induced senescence [116]—suggests that the use of chloroquine in association with senescence inducers should be considered in the clinic.

Interestingly, as cancer cells could recover from senescence and senescent cells secrete soluble factors that promote tumor growth [117], the use of drugs that selectively kill senescent cells (known as senolytics), such as the BCL-xL inhibitor navitoclax, in combination with senescence inducers and chloroquine should be a highly effective anticancer strategy against both LKB1-proficient and defective cancers.

## 6. Exploiting Selective Vulnerabilities in LKB1-Defective and LKB1-Proficient Tumors

A great effort focused on the identification of novel potential therapeutic targeting in highly aggressive *LKB1*/*KRAS* comutated NSCLC. Kim and colleagues tested 230,000 synthetic small molecules in a panel of 91 lung cancer-derived cell lines, identifying coatomer complex I (COPI) as necessary for the survival of *LKB1*/*KRAS* double mutant NSCLC. COPI is involved in the acidification and maturation of lysosomes, essential organelles in the maintenance of proper mitochondrial function. In fact, LKB1 inactivation and KRAS activation drive dependency on autophagy to fuel the Krebs cycle with carbon sources [118]. These interesting findings imply that autophagy inhibition through chloroquine, which blocks lysosome acidification, could be highly effective in killing *LKB1*/*KRAS* comutated NSCLC cells through the induction of mitochondrial dysfunction. Notably, although chloroquine has been tested in some studies aimed at targeting NSCLC [119–122], no reports in the literature refer to *LKB1*/*KRAS* mutations as a patient stratification criterion for the treatment of NSCLC.

Deoxythymidilate kinase (DTYMK) silencing has been identified as synthetically lethal with LKB1 loss in *LKB1*/*KRAS* double mutant NSCLC [123]. DTYMK catalyses the conversion of deoxythymidine monophosphate (dTMP) to deoxythymidine diphosphate (dTDP) and plays a fundamental role in nucleotide synthesis. Liu and colleagues demonstrated that LKB1 loss is associated with deficits in nucleotide metabolism. DTYMK inhibition in *LKB1*-mutated NSCLC cells leads to dUTP misincorporation in DNA, thus blocking replication. As dTMP derives from folate cycle-mediated conversion of deoxyuridine monophosphate (dUMP), hypersensitivity of *LKB1*-mutant tumors to antifolates, such as pemetrexed, raltitrexed, or pralatrexate, can be speculated. To the best of our knowledge, therapeutic efficacy of antifolates in *LKB1*-mutant lung cancer has not been evaluated in patients so far.

Another selective vulnerability in *LKB1*-mutated cancer cells is related to endoplasmic reticulum (ER) stress. Pharmacological induction of ER stress in *LKB1*/*KRAS* double mutant cancer cells triggers proapoptotic unfolded protein response and ROS-induced cell death [124]. HSP90 inhibitors and the proteasome inhibitor bortezomib are ER stress inducers currently used in the clinic. Cron and colleagues observed that proteasome inhibitors radiosensitize *LKB1*/*KRAS* double mutated NSCLC cell lines [125]. However, radiosensitization by bortezomib is a consequence of the accumulation of damaged proteins, which likely occurs independently from LKB1 status. It was observed that inactivation of LKB1 is associated with increased sensitivity to the HSP90 inhibitor 17-AAG [126–128]. Unfortunately, HSP90 chaperone protects LKB1 from proteasomal degradation [129], raising safety concerns about the exposure of normal cells to HSP90 inhibitors. Consequently, only bortezomib is a safe ER stress inducer, and more efforts should be devoted to the investigation of its efficacy in *LKB1*-mutated cancers.

Given the role of LKB1 in the maintenance of genomic integrity through the regulation of homologous recombination, its inactivation sensitizes cancer cells to PARP inhibitors [19]. PARP-1 is involved in the repair of single-strand breaks through the base excision repair (BER) pathway [130]. Ablation of PARP leads to the conversion of single-strand breaks to double-strand breaks during DNA replication, inducing cell death in homologous recombination-defective *LKB1*-mutated cancer cells. PARP inhibitors are promising anticancer drugs, some of which have been approved by the Food and Drug Administration (FDA) for the treatment of *BRCA*-mutated cancers. The use of PARP inhibitors in *LKB1*-mutated human cancers holds promise of therapeutic efficacy.

Some evidence suggests that LKB1 loss is involved in the upregulation of antiapoptotic proteins of the B-cell lymphoma 2 (BCL-2) family [131, 132], implying mitochondrial priming in LKB1-defective cancer. In particular, the activation of mTORC1 in LKB1-defective tumors drives the overexpression of myeloid cell leukemia 1 (MCL1) [62, 133]. In the last decade, a novel class of drugs, BH3 mimetics, was developed. BH3 mimetics mimic the structure of BH3 domain in BCL-2 family proteins, thus displacing proapoptotic BH3-only proteins from antiapoptotic proteins and inducing apoptosis. The upregulation of antiapoptotic proteins of the BCL-2 family following LKB1 loss suggests that LKB1-defective cancers could be sensitive to BH3 mimetics, particularly to MCL1 inhibitors, some of which—such as AZD5991—are currently in clinical trials for the treatment of hematological malignancies. The combination of MCL1 inhibitors with the BCL-2 specific inhibitor venetoclax should be effective against *LKB1*-mutated cancers and should induce a pronounced sensitization to standard chemotherapy.

Additional vulnerabilities in LKB1-defective cancers are even more speculative. The increased activation of NF-*κ*B and STAT3 pathways due to LKB1 loss could drive sensitivity to NF-*κ*B and STAT3 inhibitors in clinical trials, such as TAS4464 and TTI-101, respectively. Inhibition of these pathways should increase mitochondrial fragmentation and sensitivity to conventional therapies.

In contrast, figuring out selective vulnerabilities in LKB1-proficient cancers is not obvious. However, autophagy inhibition seems to be the most promising strategy to target drug resistance following AMPK activation, as mentioned above. In fact, the central role of ULK1 phosphorylation in the induction of angiogenesis, in the clearance of damaged mitochondria, and in maintenance of mitochondrial metabolism provides the rationale of targeting VPS34 kinase, whose activity is promoted by ULK1-mediated phosphorylation of Beclin-1. SAR405, a recently identified specific inhibitor of VPS34 kinase activity, inhibits fusion of late endosomes with lysosomes and autophagosome formation, exerting synergistic anticancer activity with the mTOR inhibitor everolimus in renal cancer cell lines [134]. Inhibition of autophagosomes leads to the accumulation of damaged and dysfunctional mitochondria, increasing the accumulation of mitochondrial ROS and inducing cell death [135]. Autophagy inhibition in LKB1-proficient tumors can be achieved with chloroquine, with some anticancer effects. However, blockade of lysosomal acidification does not impede engulfment of mitochondria in autophagosomes, which results in isolation of damaged mitochondria from the mitochondrial network. Moreover, ROS produced by damaged mitochondria inside autophagosomes must overcome two lipid membranes to reach the cytosol; thus, engulfed mitochondria release less ROS than free mitochondria.

Activated AMPK phosphorylates NRF2, thus promoting its nuclear accumulation [136]. The resulting activation of an antioxidant program is responsible for the resistance to oxidative stress observed in LKB1-proficient cancers. In fact, NRF2 activates the transcription of genes involved in the production of NADPH and induces cytoprotective autophagy [137]. Speculatively, pharmacological NRF2 inhibition should revert the resistance of LKB1-proficient tumors to ROS-inducing therapies, increasing lipid peroxidation, DNA damage, loss of mitochondrial membrane potential, and mitochondrial fragmentation, ultimately leading to cell death.

In conclusion, amongst several vulnerabilities affected by LKB1 status, dependency on cytoprotective autophagy and on NRF2-driven antioxidant response is shared by LKB1-proficient cancers and by LKB1-defective cancers driven by additional genetic alterations (i.e., activation of KRAS and loss of KEAP1).

## 7. Concluding Remarks

In the era of personalized medicine, the key role of LKB1 as a central sensor of stress opens new possibilities to target cancer cell metabolism, with important clinical implications.

The precise definition of LKB1 status represents a challenge for patient stratification. A comprehensive approach considering genetic, epigenetic, and LKB1 protein expression analysis should be taken into account.

Cancer cell metabolism is plastic and adaptable, and LKB1 plays a central role in its modulation (Figure 1). Several evidences pointed out its contextual oncogenic and tumor suppressor role. Moreover, a key function of LKB1 in modulation of tumor microenvironment is emerging. LKB1 loss is associated with a metabolic deregulation (Figure 2) that could be exploited from a therapeutic point of view.

Therefore, a better understanding of the pathways presided over by LKB1, through metabolomics and proteomics analyses, together with LKB1 status evaluation, is required to develop personalized treatment strategies. Such an approach could help to unravel the heterogeneity of cancer and to identify concurrent pathway alterations which could be targeted to overcome acquired resistance to molecular targeted therapies.

## Figures and Tables

**Table 1:** Mitochondrial dynamics control by LKB1-AMPK.

Target	Role of LKB1	Biological effects
(a) Role of LKB1/AMPK in mitochondrial fission		
MFF (mitochondrial fission factor)	AMPK-mediated phosphorylation	MFF phosphorylation relocalizes the cytosolic GTPase Dynamin-Related Protein 1 (DRP1) to mitochondria, leading to mitochondrial fragmentation [138]
ULK1 (Unc-51-Like Autophagy Activating Kinase 1)	AMPK-mediated phosphorylation	ULK1 phosphorylation initiates mitophagy of damaged mitochondria, providing cancer cells with an important loophole from therapy-induced cytotoxicity [139]
PGC-1*α* (peroxisome proliferator-activated receptor gamma coactivator 1 alpha)	AMPK-mediated activation	Activation of PGC-1*α*, the master regulator of mitochondrial biogenesis, promotes the biogenesis of new mitochondria, in order to preserve mitochondrial network functionality [139]

(b) Role of LKB1/AMPK in mitochondrial fusion		
MFN1 (Mitofusin-1)	AMPK-mediated upregulation	MFN1 mediates outer mitochondrial membrane fusion, protecting cells from mitochondrial dysfunction following a cytotoxic injury [140]
OPA1 (Optic Atrophy 1)	AMPK-mediated upregulation	OPA1 mediates inner mitochondrial membrane fusion, protecting cells from mitochondrial dysfunction following a cytotoxic injury [140]
